# Cleaning up the masses: Exclusion lists to reduce contamination with HPLC-MS/MS^☆^

**DOI:** 10.1016/j.jprot.2013.02.023

**Published:** 2013-08-02

**Authors:** Kelly Hodge, Sara Ten Have, Luke Hutton, Angus I. Lamond

**Affiliations:** a Centre for Gene Regulation and Expression, College of Life Sciences, University of Dundee, Dow street, Dundee DD1 5EH, United Kingdom; b School of Computer Science, North Haugh, University of St Andrews, St Andrews, Fife KY16 9SX, United Kingdom

**Keywords:** HPLC-MS/MS, high pressure liquid chromatography-tandem mass spectrometry, ACN, acetonitrile, PEG, polyethylene glycol, BSA, bovine serum albumin, PTM, post translation modification, MS, mass spectrometry, MRM, multiple reaction monitoring, SRM, selective reaction monitoring, CID, collision induced dissociation, *m*/*z*, mass-to-charge ratio, SILAC, stable isotope labelling by amino acids in cell culture, IP, immuno-precipitation, Contamination, Data analysis, Exclusion list, MS optimisation

## Abstract

Mass spectrometry, in the past five years, has increased in speed, accuracy and use. With the ability of the mass spectrometers to identify increasing numbers of proteins the identification of undesirable peptides (those not from the protein sample) has also increased. Most undesirable contaminants originate in the laboratory and come from either the user (e.g. keratin from hair and skin), or from reagents (e.g. trypsin), that are required to prepare samples for analysis. We found that a significant amount of MS instrument time was spent sequencing peptides from abundant contaminant proteins. While completely eliminating non-specific protein contamination is not feasible, it is possible to reduce the sequencing of these contaminants. For example, exclusion lists can provide a list of masses that can be used to instruct the mass spectrometer to ‘ignore’ the undesired contaminant peptides in the list. We empirically generated be-spoke exclusion lists for several model organisms (*Homo sapiens*, *Caenorhabditis elegans*, *Saccharomyces cerevisiae* and *Xenopus laevis*), utilising information from over 500 mass spectrometry runs and cumulative analysis of these data. Here we show that by employing these empirically generated lists, it was possible to reduce the time spent analysing contaminating peptides in a given sample thereby facilitating more efficient data acquisition and analysis.

Biological significance

Given the current efficacy of the Mass Spectrometry instrumentation, the utilisation of data from ~500 mass spec runs to generate be-spoke exclusion lists and optimise data acquisition is the significance of this manuscript.

This article is part of a Special Issue entitled: New Horizons and Applications for Proteomics [EuPA 2012].

## Highlights

• We looked at the contribution of ‘contaminating’ peptides to MS acquisition time.
• We found that 30–50% of time was wasted on contaminant peptide sequencing.
• We tested whether the application of an exclusion list would solve this problem.
• Exclusion lists generated for specific species from > 500 mass spectrometry runs.
• Exclusion lists enabled more efficient analysis with coverage and isoform data.

## Graphical abstract

**Figure:**
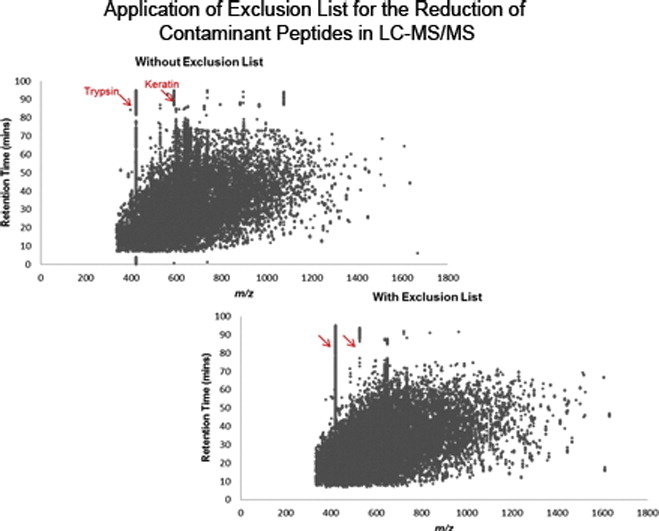

## 1 Introduction

Contamination is, by definition, the presence of either impurity, or some form of undesired, polluting substance. In biological samples contamination can take a number of different forms. For example, tissue culture cells can become infected, either with mycoplasma, bacteria and/or fungi. Instruments can become contaminated, either with oils, plastics and fibres introduced by users, or with residual traces of chemicals used for cleaning the equipment. In mass spectrometry (MS) analyses contamination is generally detected as peaks in spectra that do not originate from the protein samples to be analysed and can be introduced from a variety of environmental sources. There are two main types of contamination, corresponding to either small molecules, or to protein-related contaminants. Organic solvents like acetonitrile (ACN), detergents used for cleaning glassware and sample buffers can all introduce polyethylene glycol (PEG) into samples and thereby onto the MS peptide separation column usually coupled to the mass spectrometer. Some solvents, such as ACN, acetic acid and formic acid, can introduce unwanted metal ions (Li, Na, K and Fe^3 +^) that form adducts with other compounds. They can also give rise to phthalates (plasticizers) from plastics, such as non-low bind eppendorf tubes [1]. It is difficult to ensure the reliable elimination of these types of contaminant, due in part to the size and abundance of the molecules and to their affinity for most protein purification substrates, such as C18. As a result, good experimental technique, clean working conditions, careful sample preparation and the use of high purity solvents are the best ways to eliminate non-protein contamination.

Contaminants which are of protein origin, on the other hand, can be identified during a run and were excluded from analysis. Keratin, a fibrous structural protein abundant on the outer layer of skin, in hair and nails, is often seen as a source of contamination in MS. However, in practice limiting exposure of samples to keratin is difficult without using either a laminar flow hood, or keratin-free clean room. Keratin can also be introduced from unexpected sources. For example, a laboratory undertaking MS-based proteomic experiments started to identify sheep keratins in their samples. Investigation into this strange occurrence revealed that a member of the laboratory had started wearing a woollen jumper due to a spell of cold weather and this was the source of the sheep keratin [2]. Other common external protein contaminants arise from materials used in the experimental procedures that generated the samples. This includes proteolytic enzymes, such as trypsin (routinely added to samples to digest proteins into peptides for analysis), serum from cell media, BSA powder and casein (a sticky protein present in milk powder), used for blocking nitrocellulose membranes for western blots.

**Protein preparation quick tips**

Mass spectrometry is an invaluable analysis tool but it's only as good as the sample you provide. If the sample is poor then so is the data. Here are a few tips for good sample preparation:

➢ *Use only protein low bind eppendorf tubes.*
➢ *Wear gloves.*
➢ *Do not use schott bottles that have been to any wash up service, as they may contain polymers originating from detergents.*
➢ *Use only HPLC grade reagents (Acetonitrile, MilliQ water, Methanol, Triflouroacetic acid and Acetone).*
➢ *Keep tip boxes closed when not in use, as well as all reagent bottles and sample vials. Also never use autoclaved tips, as plastics may leach from them in higher organic solvents (i.e. ACN).*
➢ *Do protein preparation in either laminar flow hoods or in a clean, low air turbulent environment.*
➢ *When lysing cells avoid plastics if vigorous disruption is required — this will lead to polymer contamination.*
➢ *When growing material for MS analysis, maintain optimal sterility, as we have found with yeast and Caenorhabditis elegans in particular, higher amounts of keratin contamination due to growth conditions (compared to cell lines in media culture).*

As the analysis of complete proteomes becomes the focus for many studies, high levels of protein contamination can have adverse downstream effects, such as loss of low abundance proteins due to the ‘shrouding’ effect of large peaks from highly abundant peptides, under which peptides of similar mass will be lost and therefore time lost due to inefficient MS analysis.

Mass spectrometry is an extremely informative and sensitive tool for the analysis of proteins, including studies on complex cell lysates, immunoprecipitation and post translational modification (PTM) analyses [3–7]. Contamination problems affect all users of mass spectrometry facilities at differing levels. While it is virtually impossible to completely exclude contaminant peptides in a sample it *is* possible to significantly reduce the number of undesirable peptides identified in the average MS run (see ‘Protein preparation quick tips’).

Amongst the tools available to combat the problem of contamination in MS, inclusion lists, i.e., a list of specific peptide masses, can be used for a targeted analysis approach to increase the likelihood of meaningful protein identification. SRM (selective reaction monitoring) and MRM (multiple reaction monitoring) are both useful techniques, particularly for biomarker or serum analysis [8–14]. These techniques allow users to select a specific peptide precursor mass originating from the protein of interest (or masses if undertaking MRM), which is separated from a complex sample. This is achieved through the use of a triple quadrupole mass spectrometer, which is a tandem mass spectrometer that contains three quadrupoles in succession (Q1, Q2 and Q3). Q1 and Q3 act as mass filters while the centre quadrupole acts as a collision cell for collision induced dissociation (CID) of peptide fragments for further analysis in the mass analyser. This means that any peptides coming from proteins that are not required for analysis are, essentially, ignored. These techniques create a situation where only the masses for the protein of interest are analysed, but the data obtained are very specific. Unfortunately, these tools become difficult to apply when undertaking an unbiased discovery experiment, which requires all possible peptides to be identified.

**Fig. 1:**
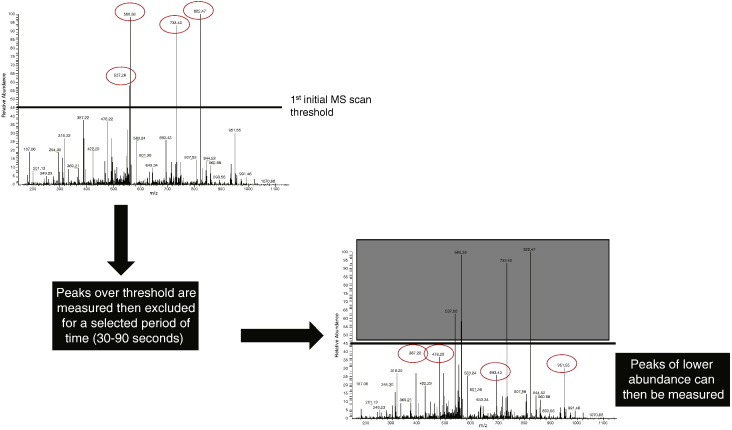
Dynamic exclusion allows the mass spectrometer to more efficiently identify peptides in a sample. The first scan measures the ions with the highest intensity (most abundant). These masses are added to a temporary ‘exclusion’ list for a period of typically 30–90 s. Once the high intensity peaks have been sequenced and excluded the MS can measure peaks under the threshold, thereby detecting less abundant peptides.

Modern mass spectrometers utilise a feature called dynamic exclusion, where a mass is temporarily placed into a list to be excluded for a selected period of time (anything from 15–600 s) [15,16], permitting the instrument to analyse other, less abundant ions (Fig. 1). It is dynamic exclusion that gives the MS the ability to ‘see’ the less abundant ions, rather than repeatedly sequencing the same, abundant peptides.

**Fig. 2:**
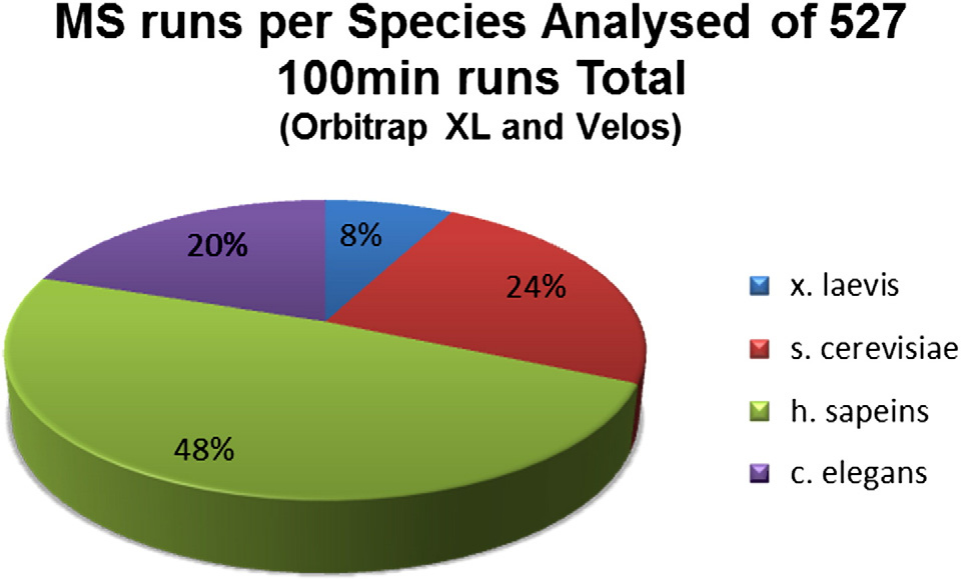
The exclusion lists were generated using 527 mass spectrometry runs. The pie chart shows the percentage of each species over the total number or runs. For *H. sapiens* 253 MS runs were used to generate the exclusion list. The *S. cerevisiae* exclusion list was generated using 127 MS runs, *C. elegans* using 105 MS runs and 42 runs for *X. laevis*. As the graph shows most of the data available to us (48%) was from *H. sapiens* reflecting the fact that most studies are performed on human cell lines.

Exclusion lists, despite the name, work with similar principles to inclusion lists. A list of masses, in this case corresponding to masses to be ignored, can be used to reduce the number of undesirable peptides analysed by the MS, whilst remaining unbiased [17–20]. Commonly this is done with an elution time associated to each mass because typically different peptides have distinct chromatographic separation properties, meaning a mass can be excluded after the time when it has been measured. We chose this latter methodology to address the problem of protein contamination. This method is preferable for large proteome coverage. To tackle the contamination problem, empirical evaluation of common contaminant peptides from widely used model organisms, including *C. elegans*, *Saccharomyces cerevisiae* and *Xenopus laevis* and mammalian cell lines, allowed us to generate be-spoke exclusion lists based on data derived from over 500 MS runs (Fig. 2).

We hypothesised that utilising be-spoke exclusion lists could reduce the number of contaminant peptides of the MS instrument sequences and thereby increase efficiency. This has been successfully applied by Muntel et. al., who generated an incremental (increasing) exclusion list for label free characterization experiments in *Staphylococcus aureus* to obtain improved data [21]. Using the incremental exclusion list approach combined with optimised mass spectrometer parameters, Muntel et. al. reported a 12% increase in additional protein identifications and accurately quantified 990 proteins (label free).

**Fig. 3:**
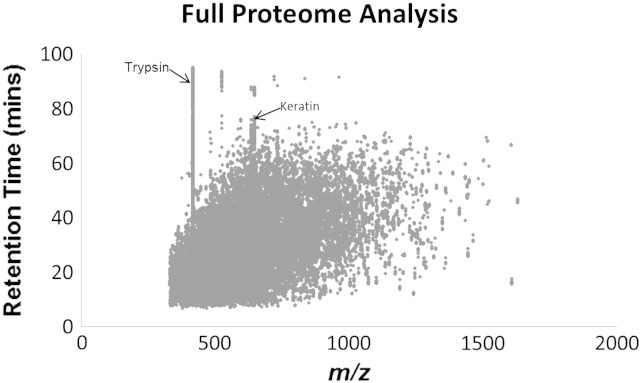
*m*/*z* (mass/charge) plotted against retention time for a whole cell proteome experiment. Contaminant peptide masses appear as vertical ‘lines’ that do not show chromatographic resolution. Peptides were identified for the digestive enzyme trypsin at 421.7584 and keratin at 769.7194.

It was during post translational modification (PTM) analysis that we realised the need to look at peptide contamination in some depth. PTM's generally occur on specific amino acids. For example phosphorylation occurs on serine, threonine and tyrosine residues, which equates to ~ 30% of PTMs in a proteome [22] meaning a full proteome analysis approach may miss the identification of phosphorylation sites. To pinpoint the location of a given modification, phosphorylated peptides need to be enriched and peptide level analysis is required. When undertaking PTM analysis, however, we observed that a large amount of instrument sequencing (MS^2^) time is spent analysing contamination peptides. Typically this can average between 30 and 50% of instrument sequencing time, despite careful sample preparation, and the application of dynamic exclusion. Fig. 3 shows a graph of mass-to-charge (*m*/*z*) ratios for all peptides detected, plotted against retention time, for a whole cell lysate. Given the chromatography applied to samples analysed by HPLC-MS/MS, distinct elution peaks for each peptide were expected. However, the data illustrated this was not the case and further investigation into the apparent lack of chromatographic resolution was conducted. The constantly eluting peptide masses were found to originate from contaminant proteins, including trypsin, keratin, casein, serum albumin and actin, all of which can be traced back to the original sample preparation and all of which have characteristically high protein concentrations within a given sample.

With large data volumes being produced by MS instruments, mass spectrometry is fast becoming a very data intensive methodology. We estimate that currently ~ 80% of the time spent on the average proteomic experiment is taken up analysing these data using appropriate software (e.g., Maxquant, Mascot Distiller, MS quant or Trans Proteomic Pipeline). This includes the time taken to extract meaningful information from the data, such as protein identifications and information concerning protein regulation. Unsurprisingly, with the ability of MS instruments to identify tens of thousands of peptides over a series of samples [23], the resulting data files are large, typically ranging from 1 to 5 Gb for the raw spectra files alone. While such large datasets can pose storage and management difficulties, they are invaluable resources that allow creation of a library of information that can be used to query datasets at a later date [24]. These libraries are extremely useful for improving the accuracy of identifying interacting proteins in IPs, for evaluating protein regulation across datasets and, in this case, for generating be-spoke exclusion lists. The data combined from numerous experiments, performed by many researchers in multiple laboratories, have been used here to generate comprehensive exclusion lists, providing an accurate coverage of the commonly identified contaminant peptides detected in the analysis of multiple human cell lines and model organisms.

## 2 Experimental procedures

### 2.1 In-gel tryptic digestion of protein samples

Full cell lysate, oligonucleotide immunoprecipitation and purified protein samples were separated by 1D SDS-PAGE using NuPAGE Bis-Tris 4–12% gels (invitrogen). In-gel tryptic digestion of samples was performed. Prior to mass spectrometry analysis, samples were cleaned using a 25 μg capacity C18 column. All protocols used can be found at http//:greproteomics.lifesci.dundee.ac.uk.

### 2.2 Mass spectrometry analysis

Peptide samples were analysed on the Velos Pro (Thermo Scientific Fisher, Boston) Orbitrap mass spectrometer coupled to an Ultimate 3000 RSLC nanoflow HPLC system (Dionex, Sunnyvale California) in the University of Dundee CLS Proteomic Facility. Sample volumes of 5 μl were eluted over a 100 min run on a 15 cm C18 column (75 μm × 15 cm nanoviper column, Thermo Scientific Boston) using a 2–40% linear gradient of solvent A (0.1% formic acid 5% ACN): solvent B (80% ACN with 0.08% formic acid) at a flow rate of 0.3 μl/min. The initial precursor scan (mass range 335–1800, resolution 60,000 and a tolerance of 10 ppm) was measured in the Orbitrap [25] and the top 15 most intense ions were further fragmented using collision induced dissociation (CID) and the MS^2^ scans were obtained. Complex lysates, immuno-precipitates and purified protein samples were run in triplicate, both with and without the exclusion list. Exclusion lists were imported as a text file format (.txt) into the precursor mass parameter in Xcalibur (Thermo Scientific Fisher, Boston, USA). Each list had no more than 2000 masses (the software limit for masses that can be excluded).

### 2.3 Bioinformatic analysis

Raw spectral data were analysed using MaxQuant version 1.0.13.13 [26–28]. The variable modifications selected were Oxidation (M) and Acetyl (Protein N-term) and Carbamidomethylation (C) as a fixed modification. Data were searched with Uniprot *Homo sapiens* database, Uniprot *C. elegans* database and Uniprot *S. cerevisiae* database release 2011_11, using the Mascot Daemon search engine (Matrix Sciences, London). The files were run with a False Discovery Rate (FDR) of 0.01% with missed cleavages set at 2 and tolerance was set at 10 ppm for MS and 0.5 Da for MS/MS.

### 2.4 Uniquences analysis

Uniquences is a native Excel add-in, written in C# that separates unique peptide sequences from large datasets. This software was used for identifying novel peptides between datasets, to determine the effectiveness of the exclusion lists. It parses input files of a pre-determined format, and extracts unique string sequences, which occur once and only once, across a number of columns, representing a distinct MS run. The add-in was written to handle files output from MaxQuant where every third column consisted of input strings of interest. When run, the program aggregates the contents of each column of interest, sorts the aggregated column, and performs a simple iterative search on each row to test whether it only occurs once in the data. If so, it is appended to a final list, which is then output back to the spreadsheet on a blank worksheet. The original design was later extended to extract additional columns of metadata from the input files. See supplementary information for installation guidelines and open source code download.

## 3 Results

To determine the magnitude of the contamination problem we analysed data from over 500 MS runs using samples from different laboratories, working on four model organisms. We visualised and compared the peptide mass data by plotting the retention time against the mass-to-charge ratio (*m*/*z*) from a complex lysate, immunoprecipitate and a purified protein dataset. The results were surprising. Despite samples being passed through a C18 separation column prior to injection into the mass spectrometer, several distinct vertical ‘lines’ appeared on the graph (Fig. 3 and supplementary data). Further investigation into the ‘lines’ identified them as peptides belonging to common laboratory contaminants (including keratins and proteolytic enzymes) that appeared to be eluting over the entire 100 min run. Table 1 presents a table of the most common contaminant proteins detected along with how often each protein was seen in samples prepared from four model organisms (*H. sapiens*, *C. elegans*, *X. laevis* and *S. cerevisae*). This empirical data were used as a starting guide for generating our be-spoke exclusion lists. Each list was then expanded by comparing all available datasets for that particular organism, resulting in 4 unique lists containing 1429, 1648, 1622 and 1029 contaminant peptide masses for, respectively, *H. sapiens*, *C. elegans*, *X. laevis* and *S. cerevisae*. Exclusion lists are included in the supplementary data.

To improve the exclusion of unwanted contaminant peptides and to provide an unbiased view of potential influences from a range of experimental approaches, we utilised data from label-free, immuno-precipitation, SILAC, time course and global proteome analysis experiments.

### 3.1 Complex lysate analysis

**Fig. 4:**
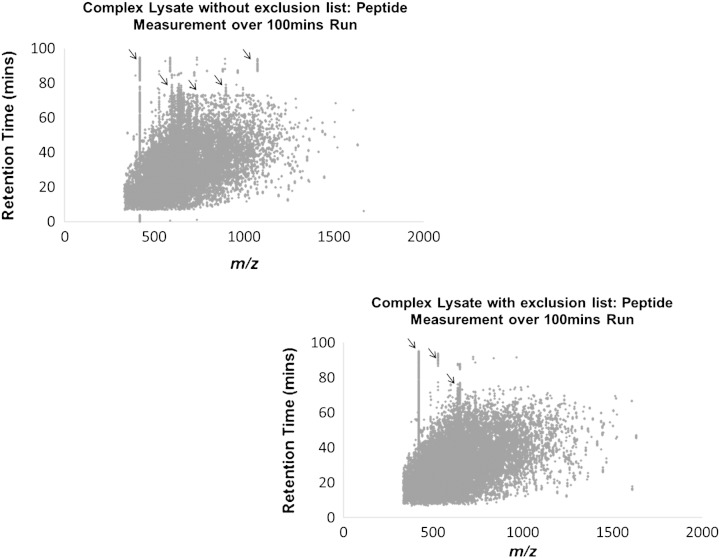
Complex lysate samples were analysed in triplicate with and without the be-spoke exclusion list. Mass-to-charge (*m*/*z*) was plotted against retention time. The first graph shows the data run without the exclusion list. The ‘lines’ are apparent in the graph that correspond to common contaminant peptide masses identified during the run such as keratin and trypsin. The second graph shows the data run with the exclusion list. While some ‘lines’ are still apparent, they appear to have decreased in frequency. See supplementary information Fig. 4 for technical replicates.

The samples were run in the Mass Spectrometer in triplicate, with and without the exclusion list. Fig. 4 (see supplementary information Fig. 4 for technical replicates) shows the mass-to-charge ratio (*m*/*z*) plotted against the retention time. The initial graph shows the data for those samples without the exclusion list. As before, we see ‘lines’ of specific masses that appear to have no chromatographic resolution. The second graph shows the data from the same sample, re-run using the be-spoke exclusion list. While the ‘lines’ of masses do not disappear completely, they do reduce in occurrence suggesting that the detection of contaminant peptides was reduced over the 100 min run. Analysis of the dynamic exclusion efficacy over a run showed that the Xcalibur software is not as efficient as expected, likely because the exclusion is not functionally 100% of the time. This is apparent also in the continuous sequencing of the same masses (‘lines’) in the initial data.

A comparison between full proteome data either with, or without, an exclusion list provided intriguing results. When samples were analysed without an exclusion list over the standard 100 min run we saw an average of 12,705 contaminant peptides sequenced. Samples run with an exclusion list were identified on average 9988 contaminant peptides. We also saw a slight decrease in the number of reverse hits (matches against a nonsense database), reducing from 68 matches to 59. This is likely due to a smaller number of spectra being acquired and therefore the proportion of those spectra being rubbish spectra (or un-assignable due to lack of known peptide sequence) and assigned to nonsense database entries is reduced, proportionally as with the contaminant peptides. We also identified 59 additional new proteins (including some additional isoform information suggesting higher sequence coverage) when using the exclusion list and improved the efficiency (from 71% to 85%).

$$\begin{array}{l} \mathrm{Efficiency}=\frac{\mathrm{Number}\;\mathrm{of}\;\mathrm{Peptides}\;\mathrm{of}\;\mathrm{interest}\;\mathrm{sequenced}}{\mathrm{Total}\;\mathrm{number}\;\mathrm{of}\;\mathrm{peptides}\;\mathrm{sequenced}*}\\ {}*\mathrm{Contamination}\;\mathrm{peptides}\;\mathrm{and}\;\mathrm{peptides}\;\mathrm{of}\;\mathrm{interest}. \end{array}$$

Over the whole proteome various unique proteins, including Arf, an ADP ribosylation factor (Q10943) and the microtubule subunit tubulin (P91910) were identified that were not seen in the initial data. Both these proteins lie outwith the normal distribution of protein abundance (top 10% PaxDB.org [29]) but were not seen in the original search, illustrating the benefit of applying the exclusion list technique to this type of data set.

### 3.2 Immuno-precipitation

**Fig. 5:**
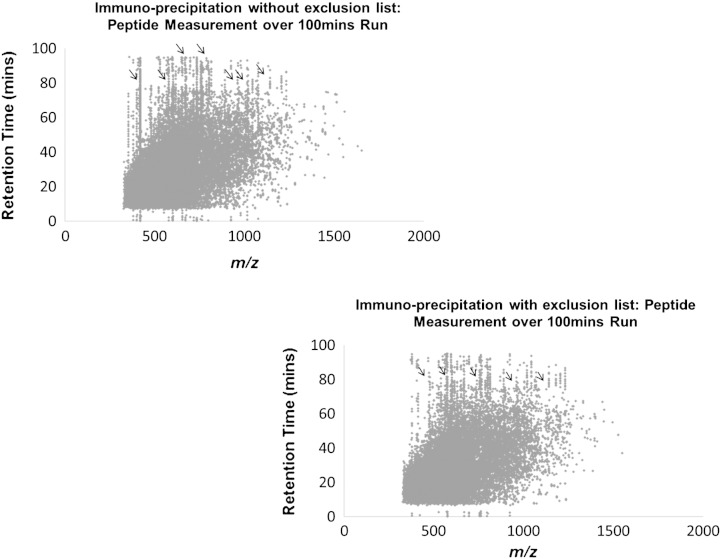
Immuno-precipitation samples were analysed in triplicate with and without the exclusion list. The *m*/*z* was plotted against the retention time. Vertical ‘lines’ of contaminant peptide masses were reduced when using the exclusion list. See supplementary Fig. 5 for the technical replicate graphs.

Analysis of lower complexity protein samples arising from immunoprecipitation experiments provided similar results. An immunoprecipitation experiment typically makes use of a specific antibody (or a specific epitope of DNA, RNA or protein) attached to a solid support that allows isolation from an extract of a protein of interest, which is specifically bound by the antibody, along with any directly or indirectly interacting proteins [30]. Immunoprecipitation experiments can be carried out at various levels of stringency, using higher salt buffers to wash away contaminants and loosely bound proteins. It should be noted however that high salt buffers risk washing away genuine interaction partners as well as contaminant proteins, especially in the case of lower affinity and non-stoichiometric binding partners. Conversely, using less stringent buffers will usually increase the number of non-specifically interacting proteins that are detected. However, using much milder wash conditions increases the confidence that fewer low abundance and/or transient interacting proteins are lost at the bench top. Consequently, using an exclusion list, in combination with a bead control sample (beads + lysate with no antibody, to characterise non-specific interactors), provides a useful approach to maximise the value of the immunoprecipitation data. The immunoprecipitate analysis shown here characterised the proteins binding to a 17 bp DNA oligonucleotide, compared with a mutant version of the same 17 bp DNA oligonucleotide sequence. As with previous data analysis we plotted the retention time against the *m*/*z* ratios for all the peptides identified over triplicate runs (Fig. 5, see supplementary information Fig. 5 for technical replicates). The ‘lines’ of specific masses were apparent in these data also. The graphs show similar trends to those seen in Fig. 4, notably, a reduction in the continually eluting ‘lines’ of contaminant masses when using an exclusion list.

This shows that using the exclusion list to aid analysis of a pull-down sample, the number of contaminant peptides identified was reduced from 6411 to 1856, a reduction of 28.9%. As seen with the analysis of the complex cell lysate data, we again identified fewer reverse hits (down from 81 matches to 44), when using an exclusion list, likely due to the acquisition of less but more specific spectra as mentioned previously. The most notable improvement, however, was the increase in instrument efficiency, which increased from 76.14% to 91.7%. This improvement allowed identification of 50 additional unique proteins that were seen only in samples run with the exclusion list. The exclusion list also allowed identification of unique isoforms of proteins, which were, again, not seen without the exclusion list. For example, during the initial run centrin 3 (O15182), an important protein involved in the microtubule-organising centre, was identified in both datasets, with or without, the exclusion list. In *H. sapiens* there are 3 different centrin proteins, Cent1, Cent2 and Cent3. While we identified centrin 3 in both datasets, centrin 2, also known as caltracin isoform 1 (P41208), was only identified when using an exclusion list. Centrin 2 has an abundance of only 1.70 ppm (parts per million), but could be identified here thanks to the use of the exclusion list.

### 3.3 Purified protein sample

**Fig. 6:**
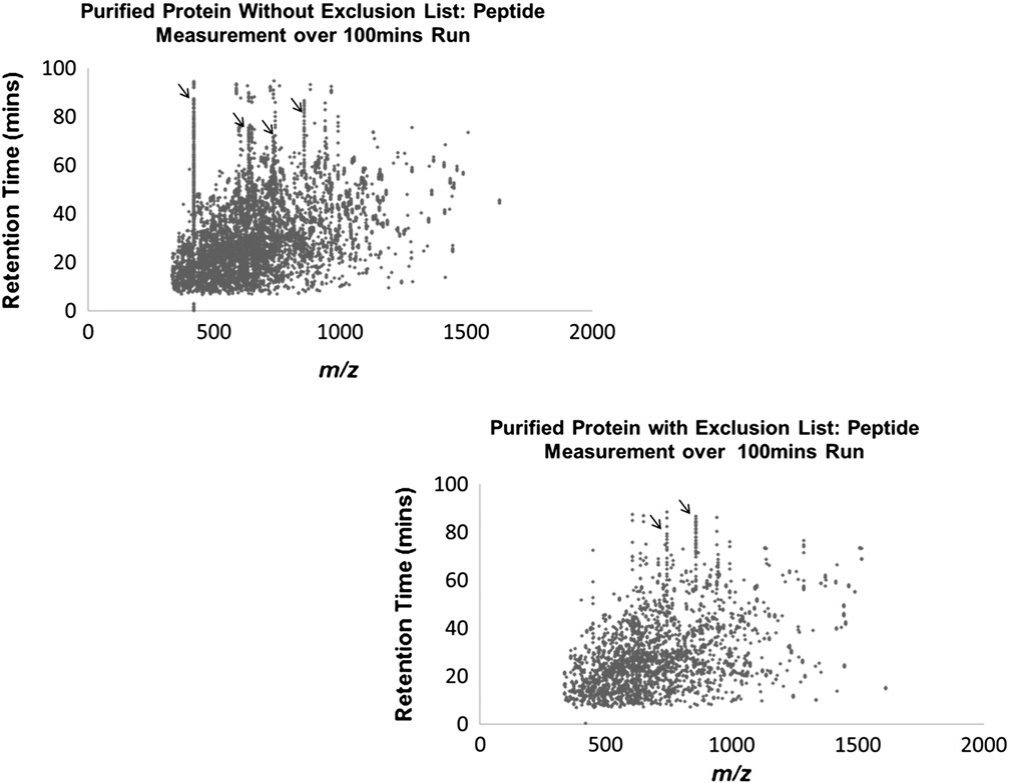
Purified protein samples run with and without the exclusion list. *m*/*z* values were plotted against the retention time. The ‘lines’ of contaminant peptide masses were apparent and these reduced in frequency when samples were run with the exclusion list. The loss of data upon use of the exclusion list is clearly visible in the lower right graph, which undermines the value of the exclusion list approach when analysing low complexity samples. See supplementary Fig. 6 for technical replicate graphs.

At the lower level of sample complexity, purified protein samples (samples which have been purified to the level of containing only the protein of interest and possibly only a few others) displayed similar trends to those seen in both the complex lysate and immunoprecipitate data (increase in efficiency from 23.7% to 54.7%), but also resulted in loss of data (peptide identifications which should occur in both sample runs were not identified with the exclusion list). This may be due to the relative simplicity of the sample, where comprehensive identification of most, if not all, of the components is feasible, due to the reduced work load for the instrument (less peptides, less MS and MS/MS required in a given time space). Giving the additional work load to exclude masses in this scenario, we conclude that for low complexity samples the use of exclusion lists may overall have a detrimental effect, as supported by the empirical data shown here in Fig. 6 (see supplementary information Fig. 6 for technical replicates). Therefore, we recommend confining the use of exclusion lists to the analysis of larger and more complex samples, such as whole cell lysates and immunoprecipitates.

We compared the abundance of the contaminant peptides for protein samples of differing complexity to test if using the exclusion list lowered the abundance of contaminants. Consistently a reduction of almost half in the abundance of contaminant proteins and peptides was seen when using the exclusion list. The reduction in abundance for contaminant peptides is likely due to the effective exclusion of these data at initial acquisition (e.g. first MS), which is the point of quantification- or intensity-measurement.

**Fig. 7:**
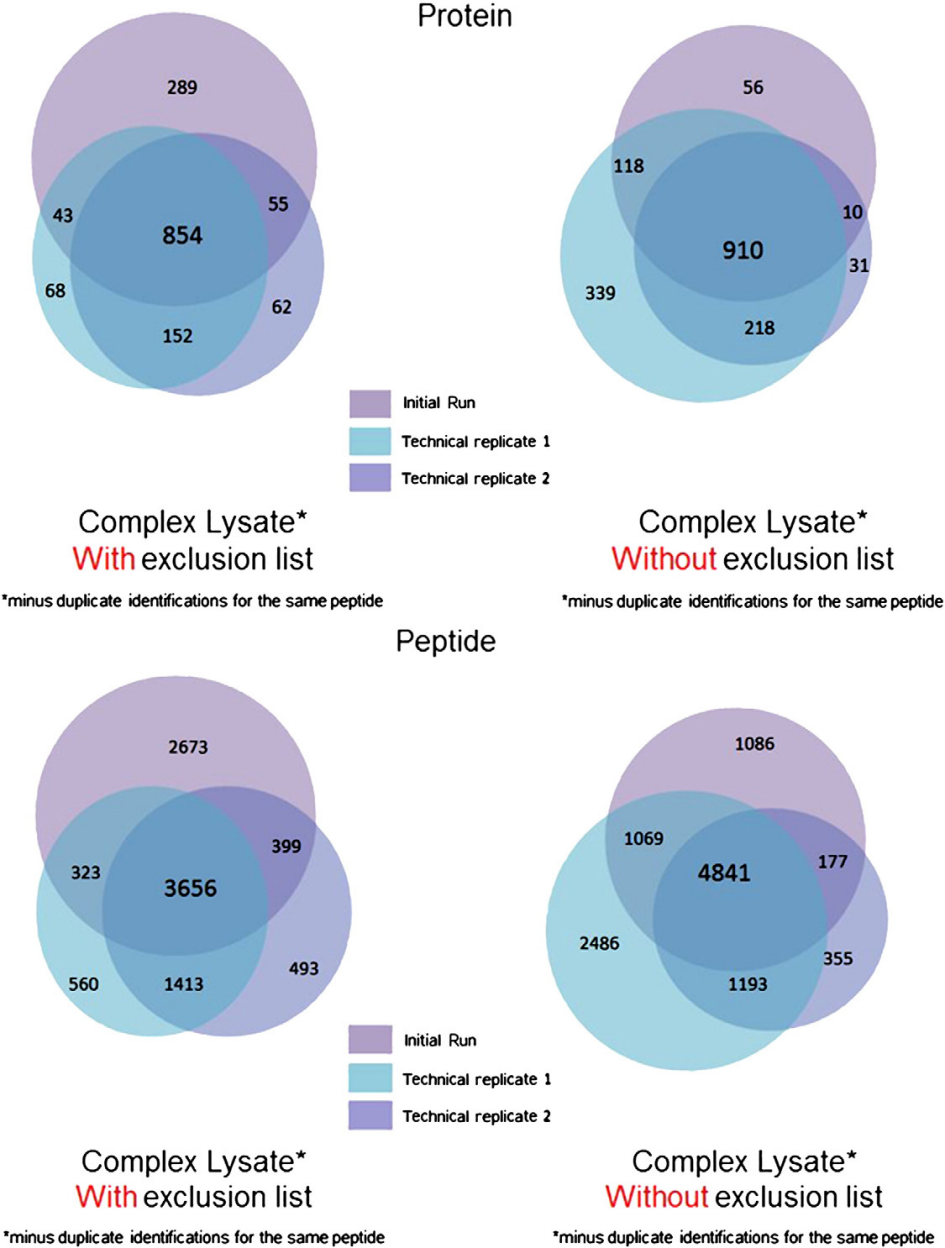
Venn diagrams showing the number of unique peptides and proteins identified for complex lysate samples run with or without an exclusion list compared against technical replicates.

**Fig. 8:**
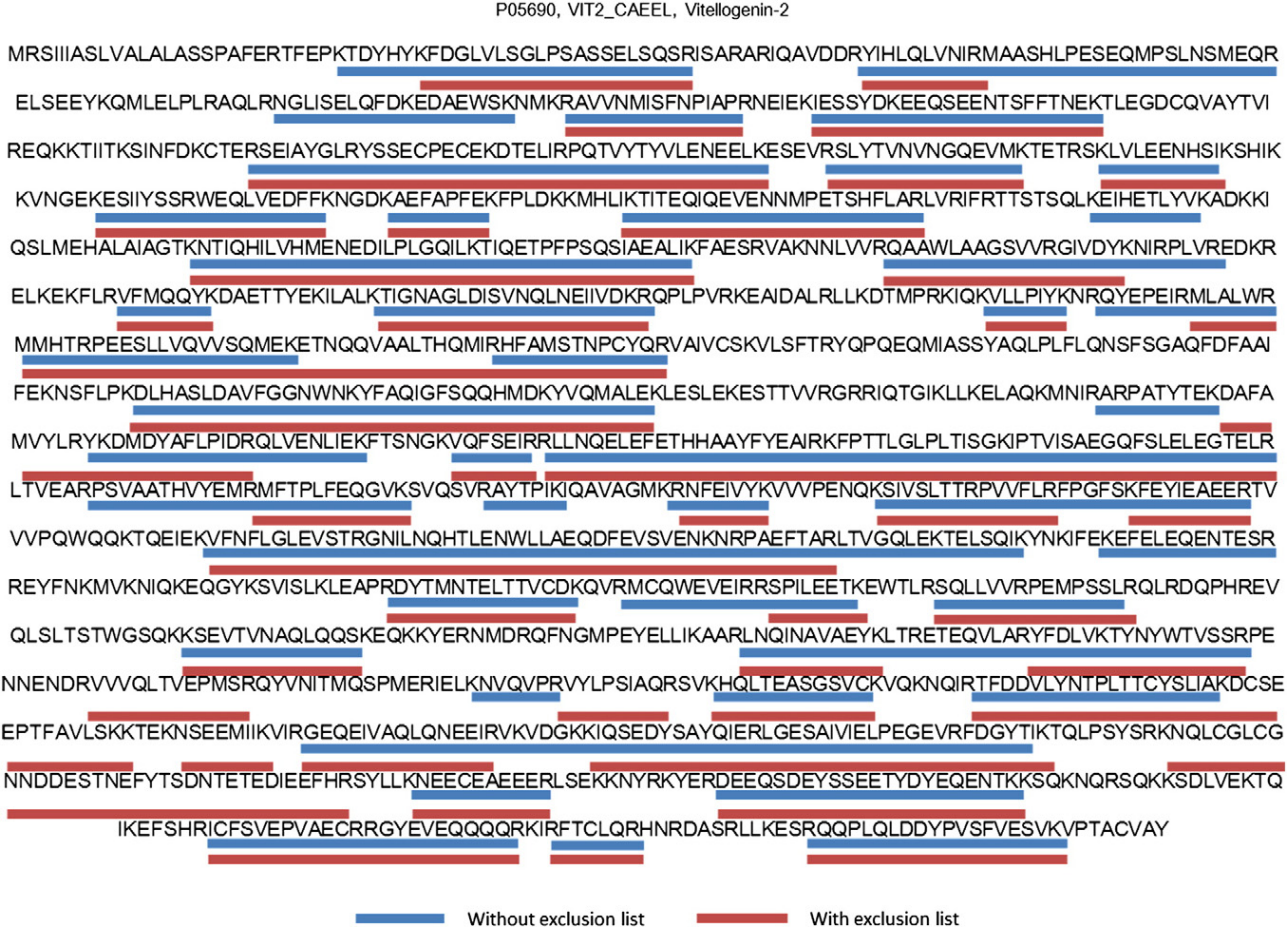
Sequence coverage diagram for the embryonic protein vitellogenin in *C. elegans*. Comparison between peptides identified during runs show that there is no loss of protein coverage when using an exclusion list.

For samples with higher protein complexity the data showed that using a be-spoke exclusion list improved the efficiency of peptide detection without any obvious drawbacks. The samples were analysed in triplicate (3 with and 3 without use of the exclusion list) to assess the contribution of any technical variance between runs. Injecting the same sample into the mass spectrometer repeatedly generally will result in additional peptide identifications. Comparing the technical replicates for a complex lysate (Fig. 7), showed that we consistently see novel peptides/proteins when using the exclusion list. While overall we see a slight decrease in the total number of unique peptides/proteins identified which coincides with the graphs previously shown (as the venn diagrams contain contaminant peptides also we expect that there will be a decrease in overall numbers with the reduction of contaminants identified). To further evaluate this we applied a bootstrap analysis on the entire complement of peptides seen (including replicate sequences) to determine if the selection of peptides in each technical replicate represented a truly random selection drawn from the same, larger, parent distribution, and to test whether there are significant differences between the data acquired with an exclusion list compared to the data acquired without. Ten thousand simulations were run and the assumption that the technical replicate data is a truly random sampling was shown to be false (given the ion selection for MS/MS is data-dependent is not surprising). No statistically significant differences between the bootstrap replicates were identified between the replicates either with, or without, the use of an exclusion list. With such low technical variance between the runs we can be confident that the differences seen with the application of the exclusion list are a result of the exclusion list, not as a result of technical replicate variance, and do not result in the loss of data. Fig. 8 shows one such example where the embryonic protein vitellogenin from *C*. *elegans* was identified in both datasets. Peptides underlined in blue were identified in samples lacking the exclusion list and those underlined in red were identified in samples with the exclusion list. While there are a few different peptides identified between the runs, the overall coverage was very similar in each case, showing that using an exclusion list did not result in loss of information.

In summary, the data show that using an exclusion list can enhance the data obtained MS analyses of medium to high complexity protein samples and increase the performance efficiency of the mass spectrometer. This was achieved by increasing protein coverage, identifying additional unique proteins and peptides (utilising ‘Uniquences’) and through identifying previously undetected isoforms.

## 4 Discussion

We have identified a number of factors that contribute to commonly observed sample contamination in mass spectrometry analyses. Unsurprisingly, a major source of contamination corresponded to various forms of keratin and other external proteins, despite taking care to prepare samples in a clean environment. It became apparent that this contamination can originate at the level of culturing the cell/organism used to conduct the experiment. For example, mammalian cell lines in culture are maintained in a clean environment, handled in a laminar flow hood and grown in sterile media, while *C. elegans* and *S. cerevisiae* can be grown at the bench top, exposed to the air, opened and analysed at the bench (away from a laminar flow hood), where they are susceptible to keratin contamination. Although all samples for proteomic analysis are susceptible to these kinds of contamination, a notable increase was seen in the model organisms grown in the conditions described above, and this should be considered when preparing these samples for mass spectrometry analysis. Cell fractionation and protein preparation protocols can differ between laboratories and even between different researchers within a laboratory and our exclusion lists aim to compensate in part for those differences.

Generally exclusion lists are not used in most routine MS analyses. To our knowledge, the applications of exclusion lists reported within the literature [31–35], usually contain both the retention time and the mass to be excluded. This is used to exclude a particular mass at the time of measurement, which is deemed to be a more efficient way of effectively excluding unwanted masses. In this study, however, we have used exclusion lists that are not linked to specific retention times because we wished to exclude contaminant peptides that may elute continuously over the entire chromatography run. Unlike previous studies [2] we also chose not to include singly charged ion species for two reasons; 1. the MS instrument used ignores all singly charged ions after the initial precursor scan (MS^1^), so the ion would not be subject to further fragmentation and analysis and 2. due to the physiochemical properties of the contaminant ions (generally our empirical exclusion list had peptide masses that had charge states between + 2 and + 4) it is more likely that they would have multiple charge states. Using the exclusion list not only helped to increase the efficiency of identification by up to two fold, but also meant that more machine time was spent sequencing meaningful information. While overall there was a slight decrease in the total number of peptides sequenced when using the exclusion list, the benefits outweigh the disadvantages. Empirically, we observe no loss of important data, indeed we see identical proteins and additional new protein ID's when comparing datasets run either with, or without, the appropriate exclusion list.

There is no reliable way to conveniently eliminate contamination from a sample completely. For example, plastic tubes *have* to be opened, thereby exposing samples to laboratory borne contamination, while bottom up proteomics (i.e., using peptide measurements to give information on proteins) requires protein samples to be digested into peptides and thus proteolytic enzymes (Trypsin, Lys-C, Chymotrypsin, and Gluc-C etc.) *have* to be added. We can, however, reduce the abundance of contaminant peptides, thereby allowing the mass spectrometer more time for relevant peptide acquisition, by using exclusion lists. It is also good practice to run samples consecutively on the mass spectrometer, both with, and without, the exclusion list. This will ensure that peptides with the same mass as any specified contaminant peptides are not excluded during the run.

We will extend this study in future by creating a library of be-spoke exclusion lists for other model organisms (*Schizosaccharomyces pombe*, *Drosophila melanogaster* and *Mus musculus*) that are widely used for proteomic analysis. We aim to facilitate the frequent use of exclusion lists that can be universally applied to a range of experimental designs with consistently reliable outcomes. Recent work into the proteomic analysis of plant extracts requires the development of additional exclusion lists that can be applied to study *Arabidopsis thaliana* and *Solanum lycopersicum* (tomato plant).

All resources required for the generation and application of the exclusion lists described in the supplementary data (exclusion lists and ‘Uniquences’) are freely available at; ‘www.greproteomics.lifesci.dundee.ac.uk’.

## Figures and Tables

**Table 1:** Table of the most common contaminant peptides identified and the occurrences (number of times peptides originating from a given protein were sequenced repeatedly) for 3 of our model organisms. A minimal threshold of over 100 peptide occurrences was set. None of the contaminant peptides from *S. cerevisiae* was above the threshold set.

	Combined total	*H. sapiens*	*X. laevis*	*C. elegans*
*Peptide count per protein*				
Keratin type II cytoskeletal 2	13,081	8448	1776	2857
Keratin, type I cytoskeletal 10	18,344	12,068	2215	3866
Keratin, type II cytoskeletal 1	12,018	832	5419	5767
Keratin, type I cytoskeletal 16	3963	2401	483	1079
Keratin, type I cytoskeletal 17	1998	1864		134
Trypsin	11,869	3320	2131	6418
Keratin, type I cytoskeletal 14	3229	2711	141	377
Keratin, type II cytoskeletal 5	4898	4285	0	613
Keratin, type I cytoskeletal 9	21,681	14,547	3724	3410
Keratin, type I cytoskeletal 6A	811	434	241	136
Serum album	476	144	186	146
Actin cytoplasmic 1	3565	0	3565	0

## Appendix A Supplementary data

Supplementary material 1. Figures 4–6 technical replicate graphs for samples run with and without an exclusion list. Installation guidelines for Uniquences, a native excel add-in is also included.

Supplementary material 2. Exclusion Lists for *H. sapiens*, *C. elegans*, *S. cerevisiae* and X. laevis.

Supplementary material 3. A test file for MS excel to ensure Uniquences is operating correctly after installation.

Supplementary material 4. An executable file that will install Uniquences as an 'Add-in' in MS excel. Uniquences appears as a separate tab on the toolbar.
