# Supplementary material for: Cleaning up the masses: Exclusion lists to reduce contamination with HPLC-MS/MS
Source: J Proteomics. 2013 Aug 2;88:92–103. doi: 10.1016/j.jprot.2013.02.023 (PMC3714598; doi:10.1016/j.jprot.2013.02.023)
Supplement: Supplementary material 1. — Figures 4–6 technical replicate graphs for samples run with and without an exclusion list. Installation guidelines for Uniquences, a native excel add-in is also included. [file mmc1.docx]

Supplementary Information: Uniquences

Uniquences

Uniquences is a native Excel add-in, written in C#. It parses input files of a pre-determined format, and extracts unique string sequences, which occur once and only once, across a number of columns, representing a distinct experimental run.

The add-in was written to handle files output from another process where every third column consisted of input strings of interest, while other columns consist of supplementary metadata (modifications and protein name respectively i.e ***sequence*** > modification > protein name > ***sequence*** > modification > protein name etc.) to help the researcher re-identify rows of data.

When run, the program copies the contents of the active worksheet to a blank worksheet. The first row, consisting of row headings, is deleted. A hashmap is populated for each column, keyed on each sequence - corresponding to an individual row - where each value is the corresponding protein name. A list of hashmaps is created, to which each column's hashmap is appended. To detect unique sequences, the program then iterates over each <key, value> pair of each hashmap. If the sequence key is found in any other hashmap, it is not unique, and the program continues to the next iteration. If the sequence is unique, the sequence is appended to a list of confirmed unique sequences.

Finally, the program switches to a third blank worksheet, into which the results of the analysis are inserted. Each unique sequence is printed to a row of the new worksheet, along with the corresponding metadata from the original worksheet to aid re-identification. Finally, the worksheet is sorted, so that sequences are displayed in ascending order, ending the operation of the program.

The original design only printed the raw unique sequences, but was later extended to extract additional columns of metadata from the input files. The program will be developed further to support arbitrary file formats, and to allow users to extend further control over the output format, so that any metadata of interest is included in the output worksheet.

To install (N/B: Uniquences is currently unavailable on the Mac platform. However as most mass spectrometry software is Windows based, to ensure compatibility with MS analysis output, the original development was done on the Windows platform, we apologise for any inconvenience):

- Download file and save to computer (My Documents).
- Run executable (.exe) file which will launch the installer.
- Uniquences will appear on the excel ribbon (N/B: you may have to re-start excel for the plugin to appear under the Peptracker tab which should now be visible).
- Run test file to ensure correct installation has been achieved. Uniquences should identify ~139 unique peptide sequences.


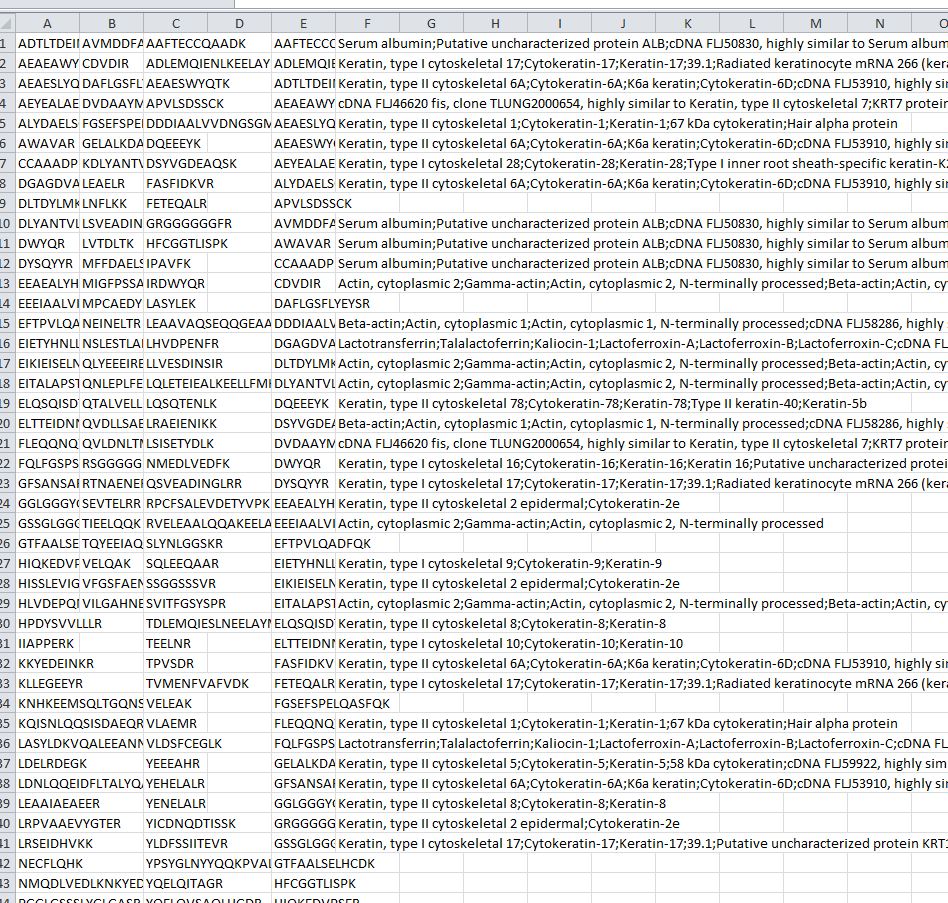
Test file output

A B C D E

Given the test file which contains 3 sets of data and thus 3 ***sequence*** columns of interest, the above output was obtained. Column A-C contains the unique peptide sequences for each individual column of interest, while column D contains a combined list of all unique peptides identified. Column E refers back to the original input file and denotes the appropriate protein name to the peptide sequence identified in D.

Supplementary Information: Technical Replicate graphs

*Figure 4*


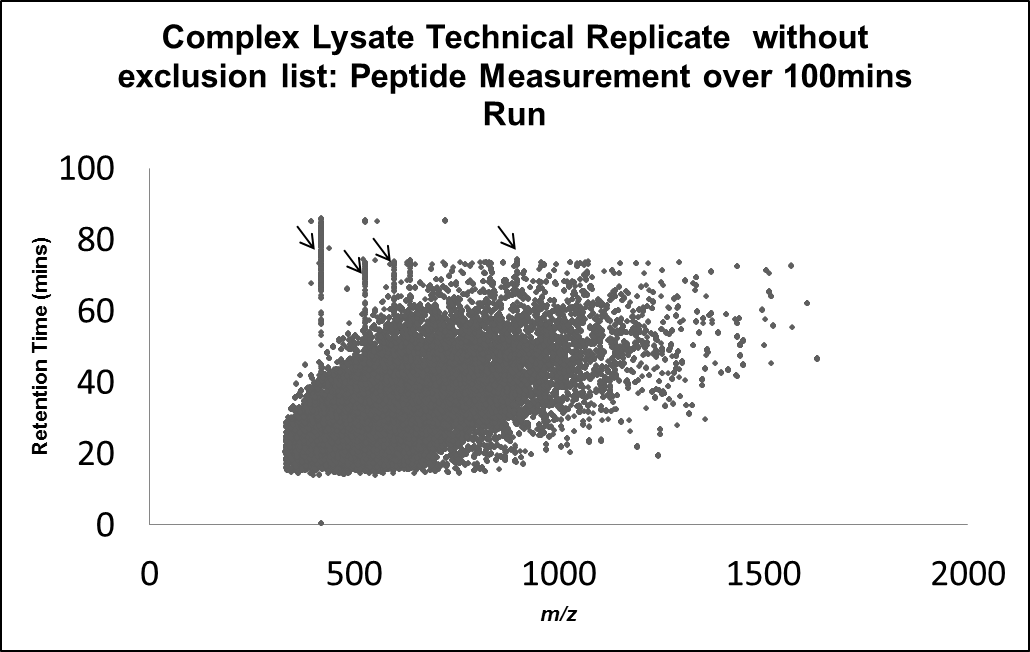

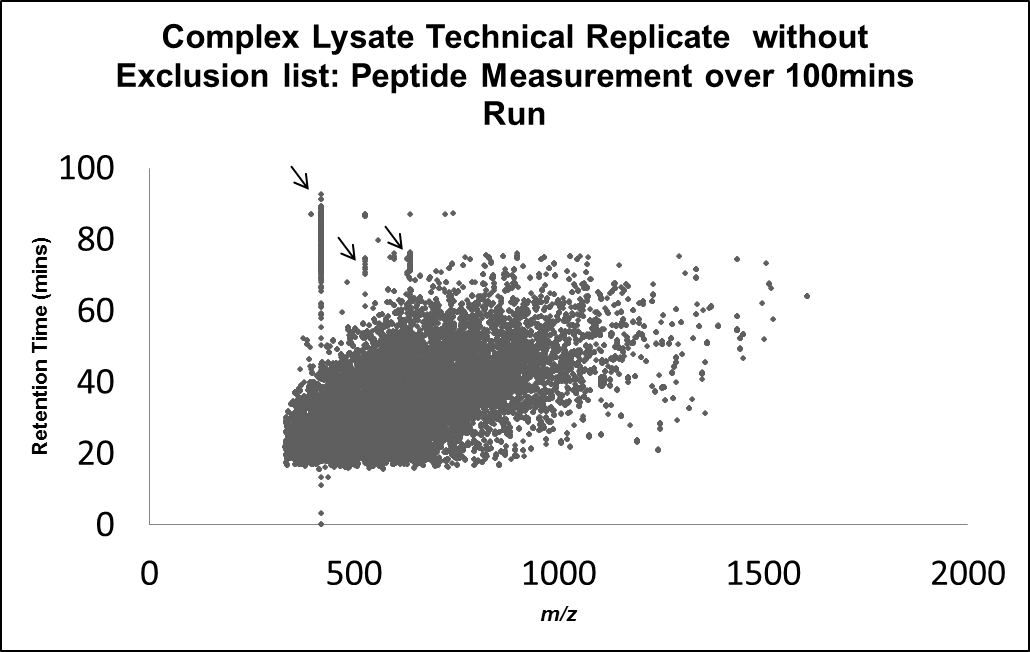

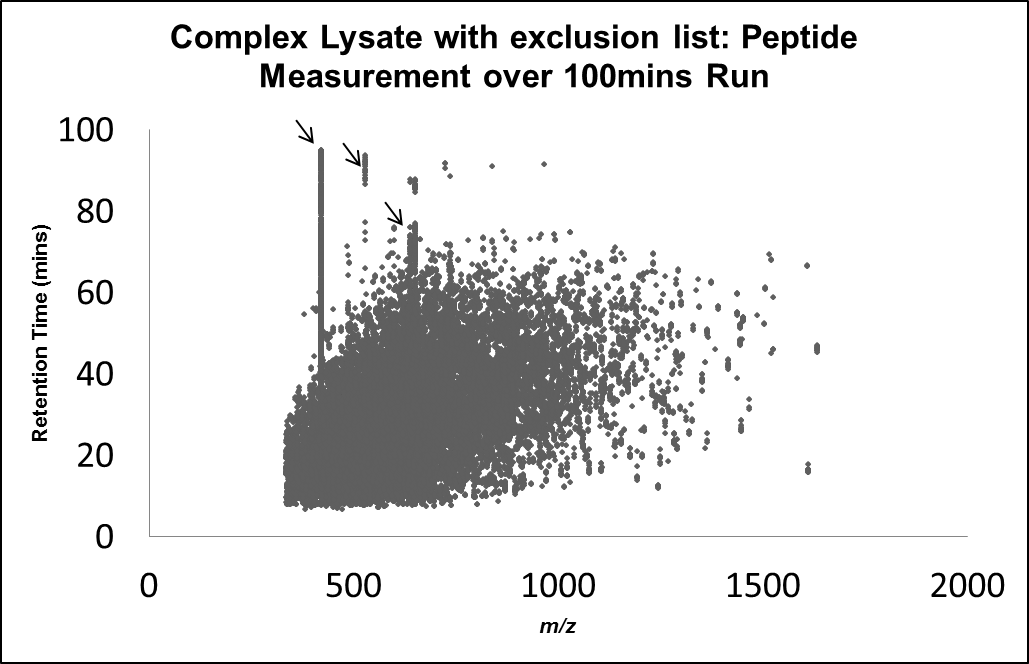

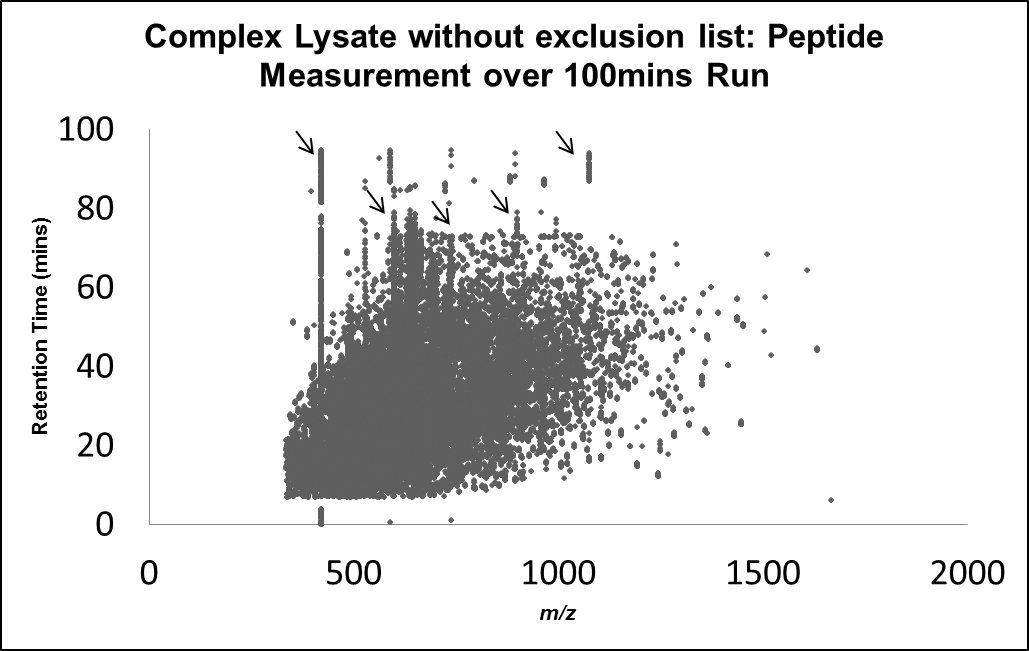

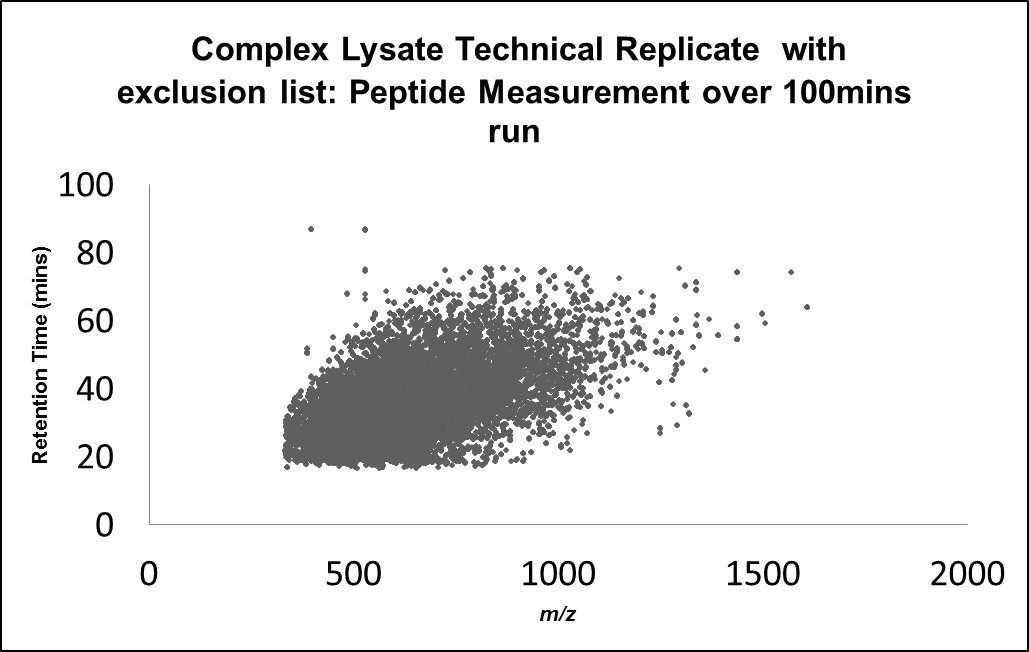

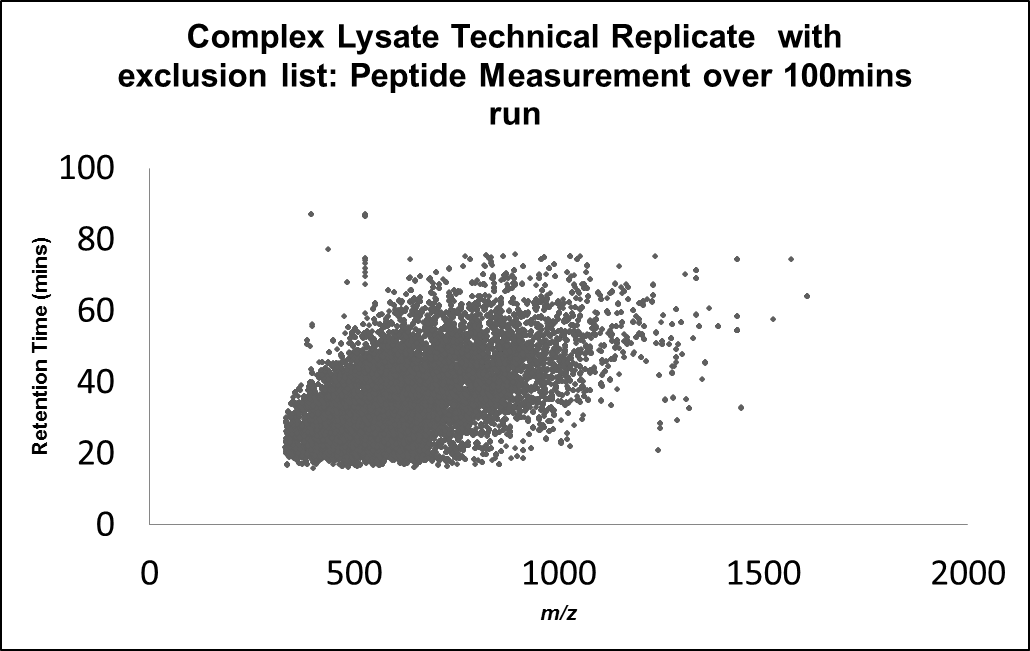


Figure 4: Scatter plots showing retention time vs. M/Z for complex lysate analysis, including technical replicates. ‘Without exclusion list’ data can be seen on the left and ‘with exclusion list’ data is shown on the right.

*Figure 5*


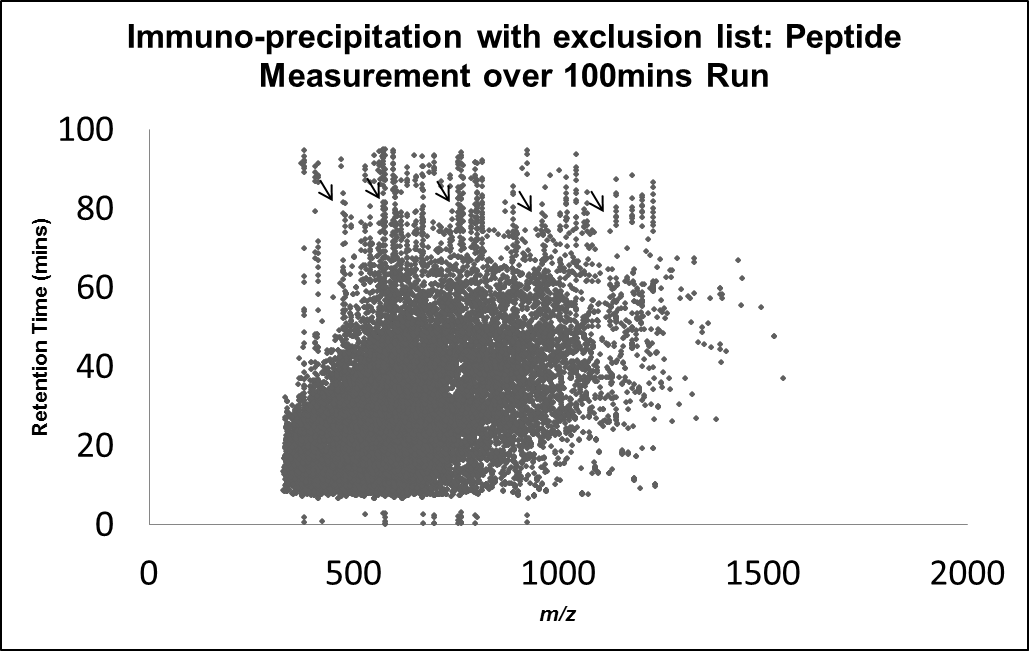

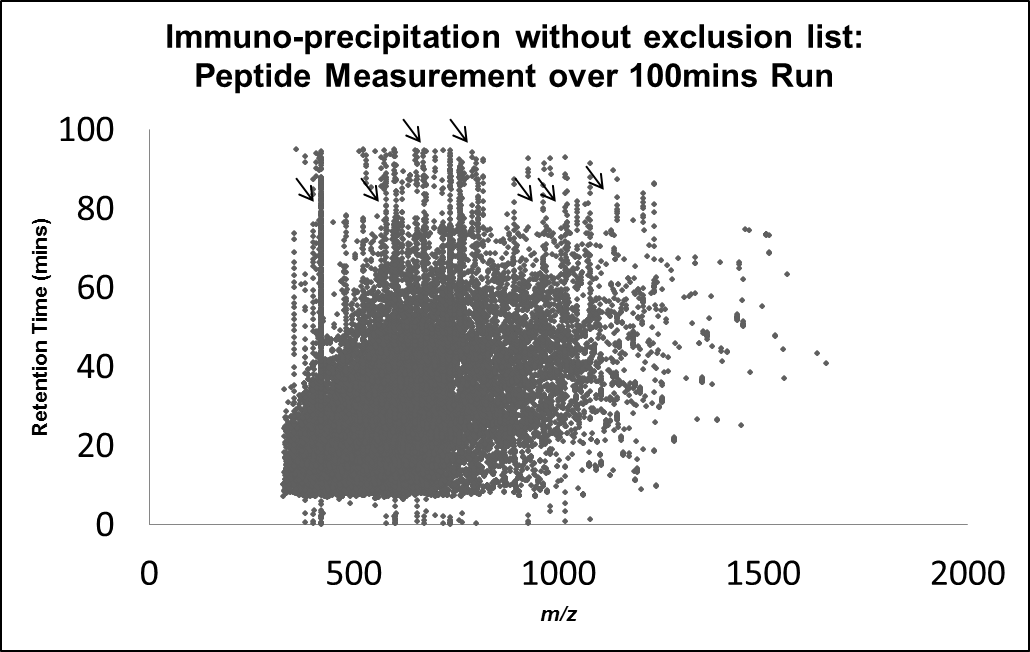

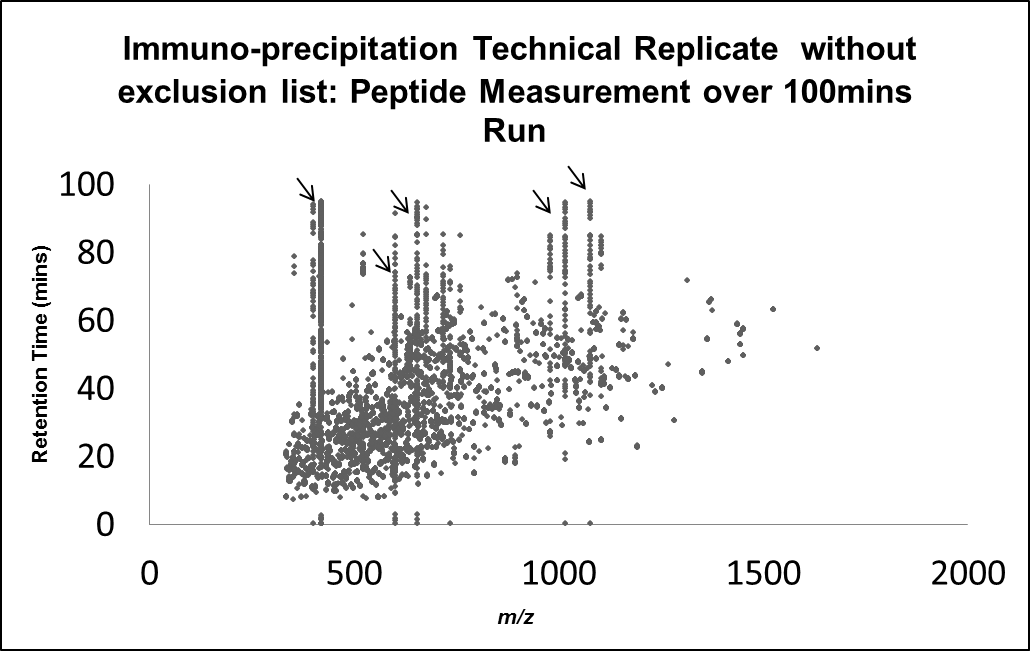

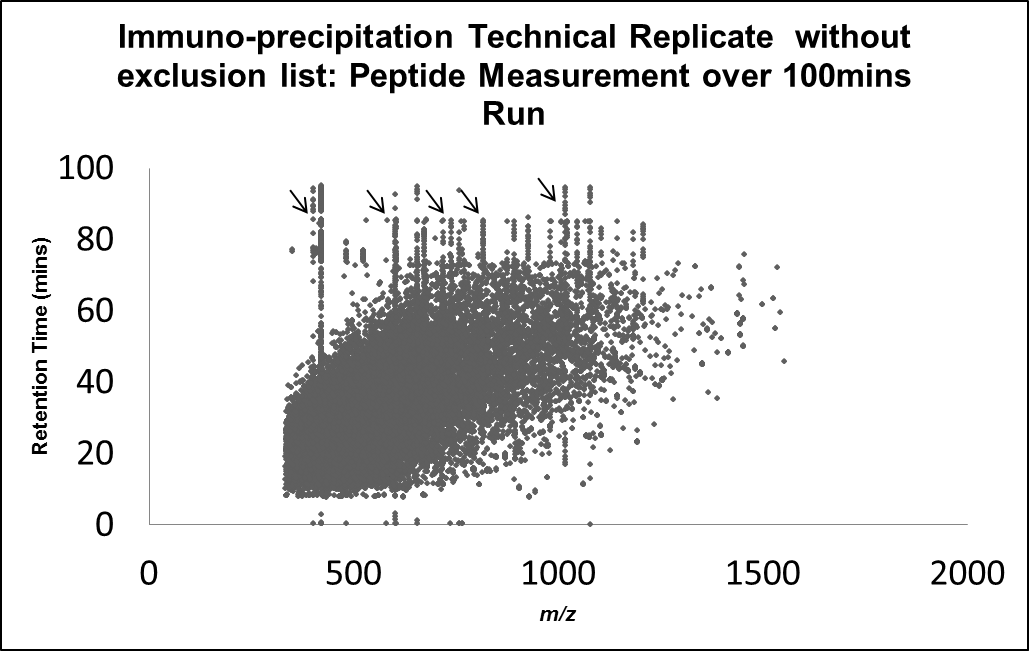

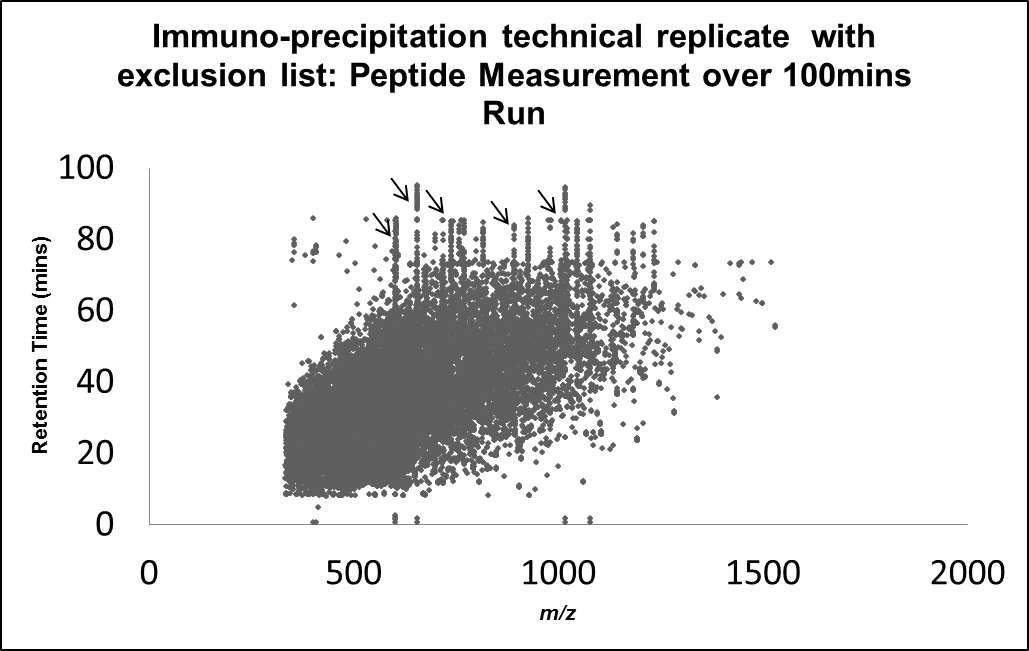

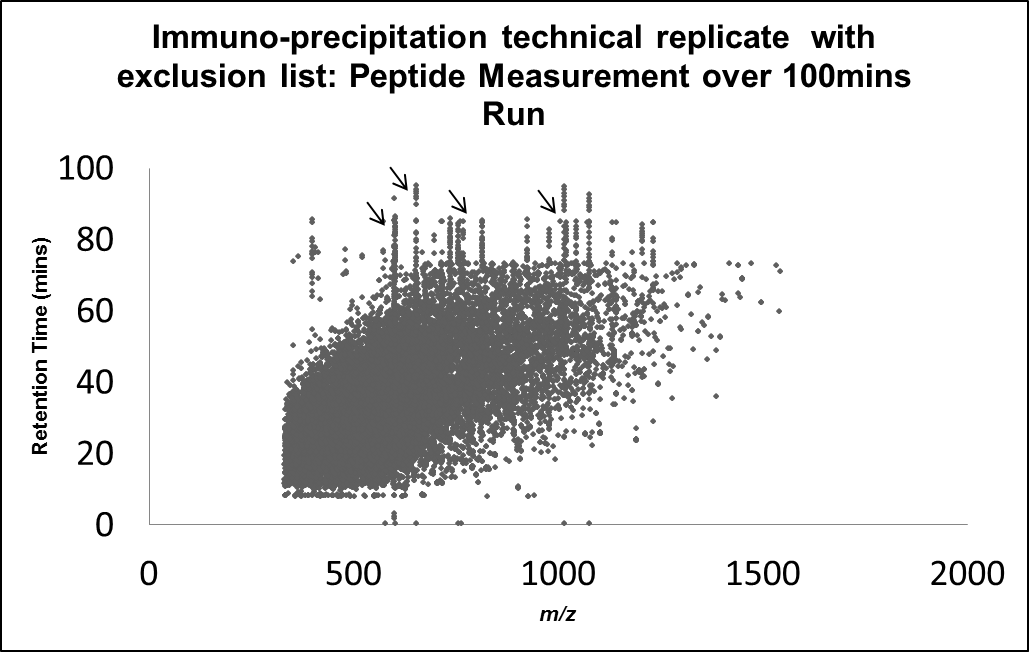


Figure 5: Scatter plot graphs showing retention time vs. M/Z for immuno-precipitation analysis, including technical replicates. ‘Without exclusion list’ data can be seen on the left and ‘with exclusion list’ data is shown on the right.

*Figure 6*


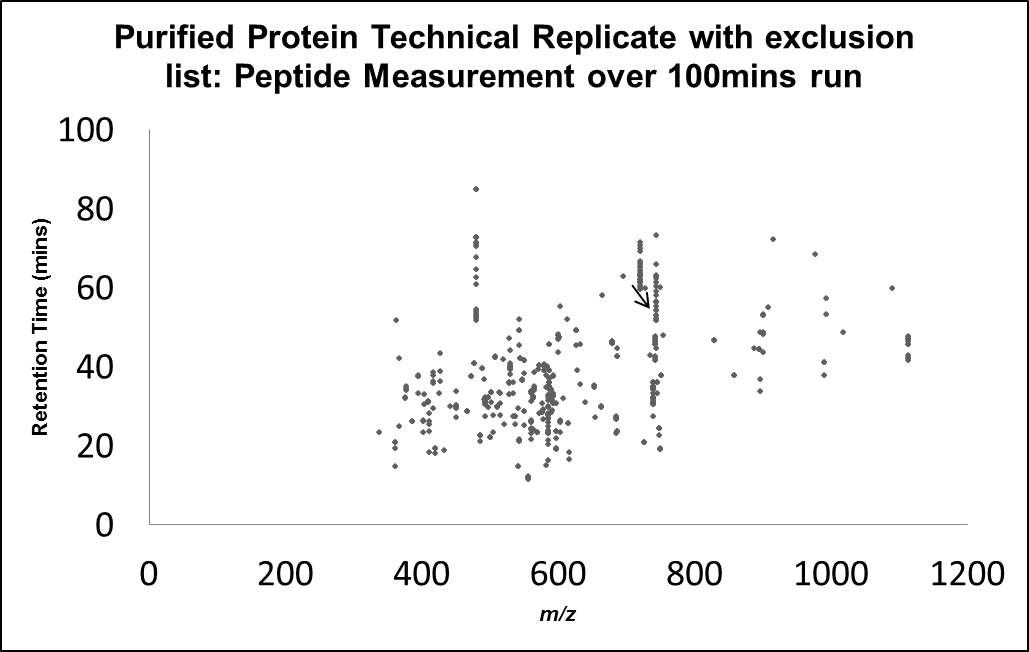

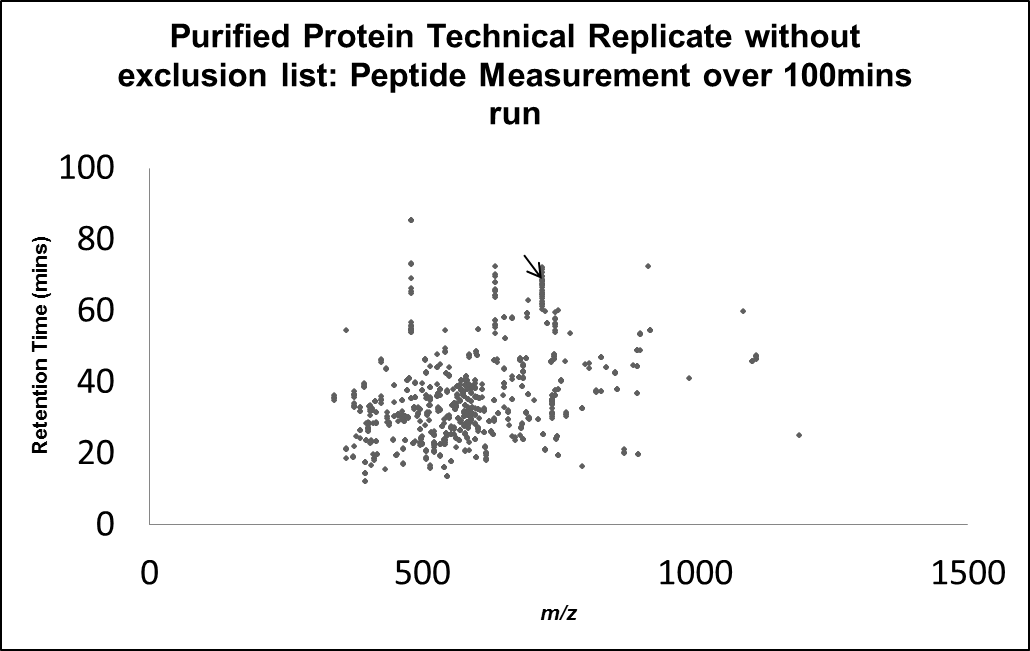

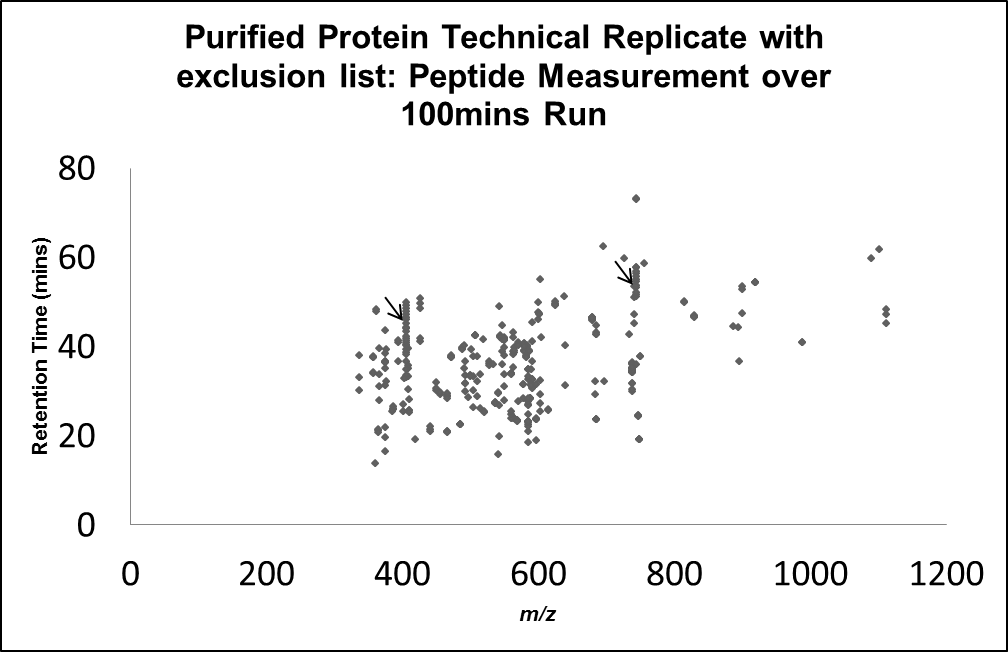

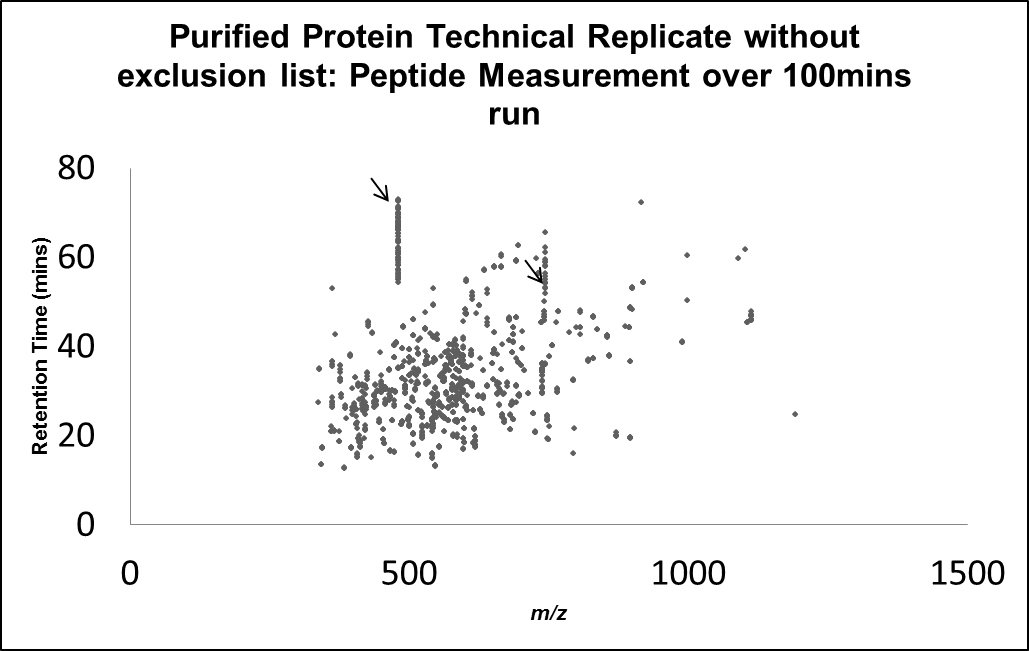

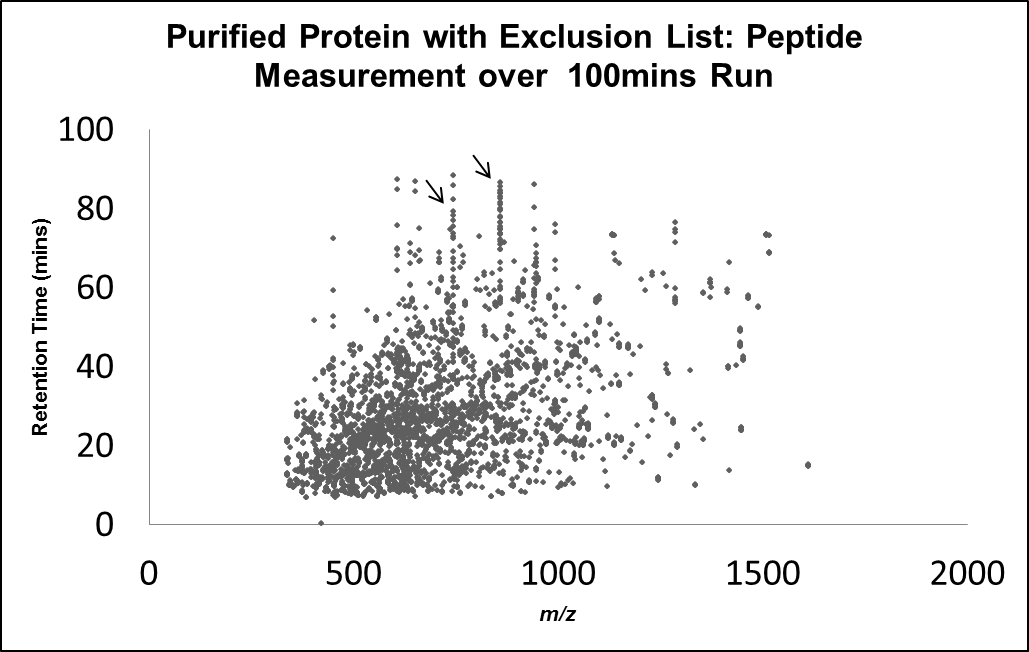

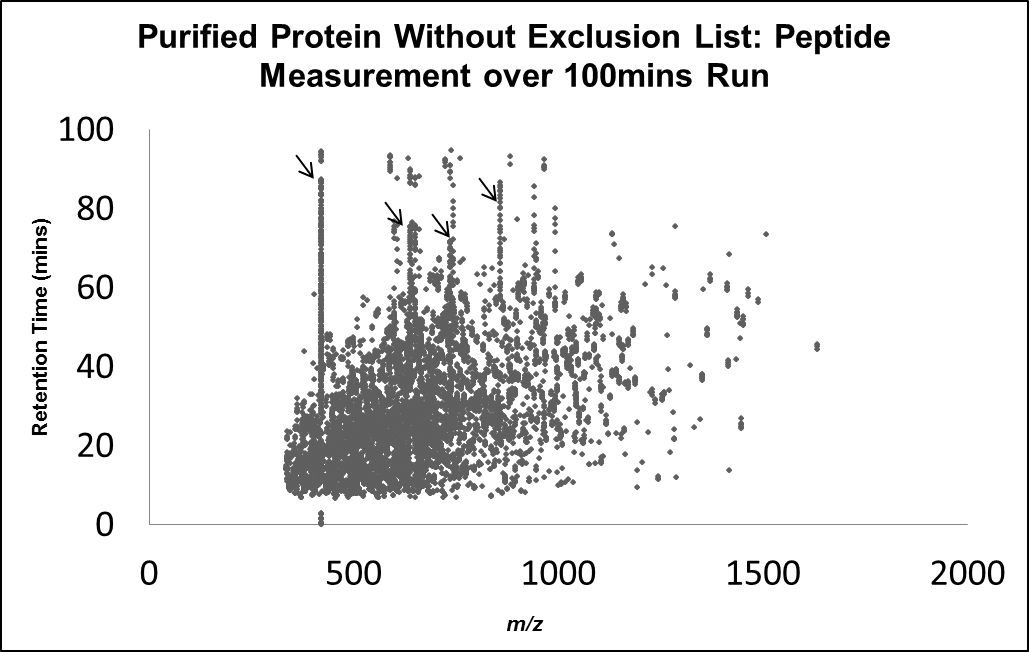


Figure 6: Scatter plot graphs showing retention time vs. M/Z for purified protein analysis, including technical replicates. ‘Without exclusion list’ data can be seen on the left and ‘with exclusion list’ data is shown on the right. The loss of data can be seen in this instance with this type of sample (purified protein) analysis, therefore we recommend an exclusion list is not necessary in this case.
